# Supplementary material for: Challenges to Cannabis sativa Production from Pathogens and Microbes—The Role of Molecular Diagnostics and Bioinformatics
Source: Int J Mol Sci. 2023 Dec 19;25(1):14. doi: 10.3390/ijms25010014 (PMC10779078; doi:10.3390/ijms25010014)
Supplement: Supplementary file 1 [file ijms-25-00014-s001.zip › ijms-2714268-supplementary.pdf]

Supplementary Table S1. Universal degenerate primers and species-specific primers to a range of viruses and virus groups that were tested in this study on cannabis leaf tissues.

357-358	Tob-Uni 1	ATTTAAGTGGASGGAAAAVCACT	10 min @ 95°C	60s @ 95°C<br>60s @ 50°C<br>60s @ 72°C
	Tob-Uni 2	GTYGTTGAGTTCATGGA		
722-723	Nepo univ A-F<br>Nepo univ A-R	ACDTCWGARGGITAYCC	10 min @ 95°C	60s @ 94°C<br>60s @ 50°C<br>60s @ 72°C
		RATDCCYACYTGRCWIGGCA		
891-892	TuRSV RNA1 Sp1F	TCGCAGGCTAGAAGAGT	10 min @ 95°C	60s @ 95°C<br>60s @ 50°C<br>60s @ 72°C
	TurSV RNA1 Sp2R	GGCCCTAAGTGTTCCTG		
783-784	Ilar1F5<br><br>Ilar1R7	GCNGGWTGYGGDAARWCNAC	10 min @ 95°C	30s @ 94°C<br>30s @ 48°C<br>30s @ 72°C<br>for 40 cycles
		AMDGGWAYYTGYTYNGTRTCACC		
749- 750	Cuc CPTALL-5 F	YASYTTTDRGGTTCAATTCC	10 min @ 95°C	60s @ 94°C<br>60s @ 48°C<br>60s @ 72°C<br>for 40 cycles
	Cuc CPTALL-3 R	GACTGACCATTTTAGCCG		
770-771-772	TobN-Univ-up3	GGCGYTGCARACIATHGTITAYCA	10 min @ 95°C	20s @ 94°C<br>15s @ 51°C<br>5s @72°C<br>for 2 cycles<br>20s @ 94°C<br>15s @ 61°C<br>5s @72°C<br>for 26 cycles
	TobN-Univ-do4	GTRTTICCIATRAAIGTIGTIACRTC		
	TobN-Univ-do4G	GCCGATRAAGGTGGTGACRTC		
846-847	AMV- F<br>AMV-coat-R	GTGGTGGGAAAGCTGGTAAA	10 min @ 95°C	45s @ 94°C<br>45s @ 50°C<br>45s @ 72°C<br>for 35 cycles
		CACCCAGTGGAGGTCAGCATT		
628-629	Potyvirus univ – Nib2F	GTITGYGTIGAYGAYTTYAAYAA	10 min @ 95°C	45s @ 94°C<br>45s @ 52°C<br>45s @ 72°C<br>for 36 cycles
	Potyvirus univ – Nib3R	TCIACIACIGTIGAIGGYTGNCC		

Supplementary Table S2. Quality characteristics of RNA isolated from seven genotypes of cannabis.

Sample ID	ng / µL	260/280	260/230
PDH	199.23	1.98	1.55
PD	184.92	2.07	1.91
Mac-1	82.07	2.10	2.20
PK	232.67	2.09	1.98
BC	115.54	2.07	1.02
CBD	77.42	2.14	2.15
G54-2	253.73	2.08	2.23
